# Ago1 is required for the regulation of mitochondrial translation under heat stress in *Schizosaccharomyces pombe*

**DOI:** 10.1016/j.jbc.2026.113235

**Published:** 2026-06-04

**Authors:** Mengling Yu, Yuanqi Xu, Yijing Lu, Minjie Li, Yucheng Yao, Gang Feng, Ying Huang, Jinjie Shang

**Affiliations:** Jiangsu Key Laboratory for Microbes and Functional Genetics, College of Life Sciences, Nanjing Normal University, Nanjing, China

**Keywords:** *Schizosaccharomyces pombe*, mitochondria, mitochondrial protein synthesis, mitochondrial translation, heat stress

## Abstract

Mitochondrial protein synthesis is a critical component of OXPHOS complexes, vital for both mammals and *Schizosaccharomyces pombe*. In our study, we investigated the effect of heat stress on mitochondria, analyzed the mitochondrial proteome and found that during heat stress, the translation of all mtDNA-encoded transcripts was impaired, leading to a reduction in the steady-state levels of mtDNA-encoded proteins, suggesting that heat stress plays a general role in mitochondrial protein synthesis. We also found that heat stress affects the association of mitochondrial translation initiation factors to mitoribosomal small subunits. Interestingly, *ago1* deletion compensates for the heat-induced disruption of the interaction between mitochondrial translation initiation factor and mitoribosomes, leading to partial recovery of both translation and steady-state levels of mtDNA-encoded proteins in *S. pombe*. Under heat stress, Ago1 accumulates in the mitochondrial matrix. C-terminal truncation ablates this localization and abolishes rescue of translational suppression, confirming mitochondrial targeting is essential for regulatory function. Furthermore, our data demonstrate that Ago1's small RNA-loading-related N-terminal domain is required for heat-induced translational suppression and that Ago1 physically engages with mitochondrial RNAs, collectively indicating potential RNA interference (RNAi) activity within mitochondria. These findings provide insight into the regulation of mitochondrial protein synthesis in heat stress.

Mitochondria are organelles that perform numerous essential cellular functions in addition to their traditionally established functions in energy metabolism. These functions are dependent on the enzymatic activities provided by many different mitochondrial polypeptides, of which the majority are encoded in the nucleus, whereas only a few polypeptides are generated by the endogenous mitochondrial protein biosynthesis machinery. Mitochondrial homeostasis is therefore dependent on a coordinated expression of both nuclear and mitochondrial genes. The mechanisms underlying this coordination process, in particular during adaptation to various stress conditions, are still only inadequately known and are an active field of cell biological research (1).

Mitochondria are energy-producing organelles in eukaryotic cells involved in adenosine triphosphate (ATP) synthesis through oxidative phosphorylation (OXPHOS) (2, 3). Besides ATP production, mitochondria participate in numerous critical cellular processes, including the biosynthesis of vitamin cofactors, amino acids, and fatty acids, as well as programmed cell death pathways such as apoptosis and autophagy (3, 4, 5). Mitochondrial dysfunction is associated with aging, cardiovascular disease, metabolic disorders, including diabetes, and neurogenerative diseases such as Alzheimer’s disease and Parkinson’s disease (4, 6, 7, 8).

Of the more than 1200 proteins present in mitochondria, only 8 (Cob1, Cox1, Cox2, Cox3, Atp6, Atp8, Atp9, and Var1) are encoded by the fission yeast *Schizosaccharomyces pombe* mitochondrial DNA (mtDNA) (9, 10). These mitochondrial-encoded proteins constitute important components of all complexes of the electron transport chain (ETC) except for complex II, the components of which are exclusively encoded by the nuclear genome (10, 11, 12, 13). Therefore, the nucleus and mitochondria must continuously coordinate the transcription, translation, translocation and import of mitochondrial proteins. Mitochondria contain their own DNA (mtDNA), which exhibits distinct characteristics from nuclear DNA, including a polyploid state and a more compact organization (10, 11, 12, 13). Similar to animal mtDNAs (∼16 kb), the fission yeast *S. pombe* mtDNA is a compact genome (∼19 kb) (10, 12, 14). *S. pombe* mtDNAs encode two rRNAs (*rns* and *rnl*), eight mRNAs (Cob1, Cox1, Cox2, Cox3, Atp6, Atp8, Atp9 and Var1), 25 tRNAs and the RNA subunit of RNase P (*rnpB*) (9, 10).

Different fungal species have different optimum temperature ranges for growth and survival. A modest increase in physiological temperature could also be beneficial for organisms under specific conditions because fever in vertebrates has a survival benefit by regulating the immune system and protecting the organism against infection. Similar to vertebrates, temperature fluctuations exert significant effects on key cellular processes in yeast. However, severe ambient temperature can disrupt growth, affect fungal biology and cause damage including misfolded and aggregated proteins, organelle fragmentation, nuclear swelling, elevated oxidative stress, and cell cycle arrest (15, 16). Fungi have developed various mechanisms to cope with damage and adapt to high-temperature changes. When the temperature increases, cells activate their heat shock response by synthesizing heat shock proteins (17). These proteins help to maintain protein stability and ensure the survival of the cell (17). Research has largely focused on the response patterns and molecular pathways of the cytoplasm to heat shock stress now. The heat shock response is controlled by heat shock transcription factors, which induce the expression of specific molecular chaperones under stress (18). However, if the protein quality control system is unable to keep up with increased demand, cells have additional protective mechanisms available. One strategy is to sequester protein aggregates into specialized compartments, while another is to degrade or refold proteins into their native conformations. These adaptive mechanisms provide protection against the harmful effects of high-temperature stress (19, 20). To improve the economic efficiency of industrial production and to combat human fungal pathogenesis, there is an increasing amount of research focused on understanding the cytoplasmic response of fungi, including *Saccharomyces cerevisiae* (21) and overall mitochondrial function (22).

Although substantial information has been obtained on the cytoplasmic response to heat-shock stress, little is known about the effects of heat stress on mitochondrial proteins and overall mitochondrial function. Previous studies conducted in *S. cerevisiae* have revealed that, at elevated temperatures, vital enzymes involved in mitochondrial metabolism tend to aggregate and subsequently become inactivated. The aggregation rate of these enzymes is regulated by mitochondrial chaperones and Pim1 protease, present in the matrix compartment (23). Interestingly, mitochondrial proteins were found to be overrepresented in a screen for aging-related protein aggregation in *Caenorhabditis elegans* (24). This finding establishes a link between mitochondrial protein aggregation and age-related disorders. Critically, research on mitochondrial adaptation mechanisms under hyperthermia remains strikingly scarce compared to cytoplasmic stress pathways, with specific regulatory principles yet to be defined. Moreover, the protein players mediating stress-adaptive translational control within mitochondria are largely unknown.

In this study, we demonstrate that heat stress triggers a global reduction in the translation of mitochondrially encoded proteins in *S. pombe*, indicating a universal regulatory principle governing mitochondrial protein synthesis. We further reveal impaired association between mitochondrial translation initiation factors and the mitoribosomal small subunit under thermal challenge. Moreover, we demonstrate that the cytoplasmic RNA interference protein Ago1 is involved in the heat-induced regulation of mitochondrial translation. Collectively, these findings offer insights into the mechanistic basis of mitochondrial proteostasis under thermal stress.

## Result

### Global proteomic combined screening identifies heat stress impairs effects on *S. pombe* mitochondrial proteome regulation

**Figure 1:**
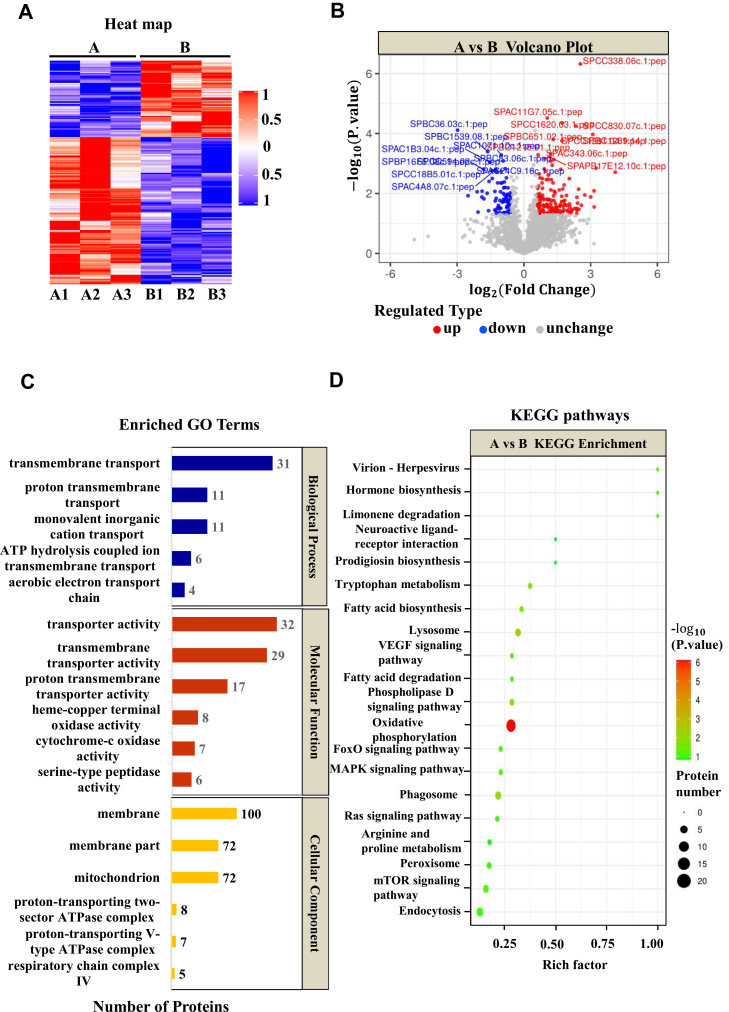
**Heat shock shows a significantly affect according to the mitochondrial proteomic data.***A*, Mitochondrial proteomics identification of WT after heat shock treatment. Group A represents unstressed wild-type strains and Group A represents heat shocked wide-type strains. Clustering of differentially expressed proteins across six samples is shown. The horizontal lines represent genes, each column is a sample, red represents high-expressed genes, blue represents low-expressed genes and gray represents no protein quantification information, expression levels of significantly differentially expressed proteins were standardized using the z-score method, with selection criteria of fold change > 1.5 and *p*-value < 0.05. Each group has three parallel samples, namely A1, A2, A3, B1, B2, and B3. *B*, Volcano plots of proteomic analysis of differentially expressed proteins between A group *versus* B group. Each point represents a protein, protein’s fold change in abundance is plotted against statistical significance (*p*-value). Log_2_FoldChange is the abscissa and -log_10_ (*p*-value) is the ordinate. *Red dots* represent upregulated proteins, *blue dots* represent downregulated proteins, and *gray dots* represent proteins with no statistically significant changes. *C*, GO enrichment analysis of differentially expressed proteins under heat shock, Blast2GO was used to annotate target protein collections with GO annotations. GO enrichment findings of differentially expressed proteins under heat shocked condition were classified as molecular function (MF), biological process (BP), and cellular component (CC). Vertical coordinates in the graph represents GO secondary level functional annotation information, *blue* represents biological process, *red* represents molecular function, *orange* represents cellular component; horizontal coordinates indicate the number of differentially expressed proteins under each functional category. *D*, KEGG pathway annotation statistics of heat shocked condition (Top20). KEGG Automated Annotation Server (KAAS) was used to execute KEGG pathway annotation on target protein datasets. The enrichment factor (Rich Factor ≤ 1) is the abscissa which quantifies the ratio of the number of differentially expressed proteins annotated to a KEGG pathway category to the number of all identified proteins annotated to that category and then lists the top 20 major pathways enriched for the KEGG pathway are the ordinate. The size of the bubbles reflects the number of differentially expressed proteins, and the color represents the significance level of enrichment. Color is based on the *p*-value from Fisher’s exact test, with the color gradient indicating −log_10_ (*p*-value).

To explore the molecular regulatory mechanisms of the mitochondrial proteome in *S. pombe* under heat stress, we performed label-free proteomic analysis to compare global protein expression between heat-shocked and untreated *S. pombe* strains cultured for 12 h. The statistical analysis of the samples in two groups was accomplished by heatmap (Fig. 1*A*) and volcano plot of differentially expressed proteins (Fig. 1*B*), cluster analysis of GO items (Fig. 1*C*) and KEGG pathway enrichment analysis (Fig. 1*D*).

In the heatmap, we used expression difference greater than 1.5-fold and *p*-value less than 0.05 as the screening criteria. The resulting significantly differentially expressed proteins could be effectively distinguished, indicating that the screening of differentially expressed proteins could represent the effects of biological treatments on the samples. (Fig. 1*A*). The results showed that the proteomic data could be used for further analysis. We have identified 3136 *S. pombe* proteins associated with mitochondria, 160 proteins were identified to be differentially expressed, and 83 proteins were significantly downregulated under heat shock. In addition, we labeled the top 10 up- and down-regulated proteins with the most significant differences each by name and separately listed the five most significantly different proteins (Fig. 1*B*).

All significantly differentially expressed proteins were tagged with GO function using Blast2Go (https://www.blast2go.com/), GO enrichment analysis of heat stress were classified as molecular function (MF), biological process (BP), and cellular component (CC) (Fig. 1*C* and Tables S3–S5). Statistical analysis revealed that heat stress-related proteins in the mitochondrial proteome differential expression profile of *S. pombe* were primarily enriched in the biological process of proton transmembrane transport. Most localized to two cellular components: the cytosolic membrane and V-type ATPase complex for proton translocation. This phenomenon may relate to temperature stress affecting cellular membrane fluidity and permeability. In addition, the mitochondrial heat shocked proteins of *S. pombe* were mainly responsible for the activation of proton transmembrane transporter activity, monovalent inorganic cation transmembrane transporter activity, and cytochrome C oxidase activity. Concomitantly, KEGG analysis revealed significant enrichment in metabolic pathways linked to oxidative phosphorylation, lysosomes, phagolysosomes, phospholipase D signaling, tryptophan metabolism, and fatty acid biosynthesis (Fig. 1*D* and Table 1). Our findings highlight not only the proteins affected by heat stress, but also the interrelation between various pathways. Specifically, we identified functional linkages among lysosomes, phagosomes, and endocytosis pathways. Notably, the phospholipase D signaling pathway is accompanied by endocytosis and the MAPK signaling pathway. This explains why there is a significant number of differentially expressed proteins in the endocytosis pathway, even though it is not highly enriched. The enrichment analysis of proteins significantly altered by temperature stress revealed the oxidative phosphorylation pathway as the most highly enriched. Interestingly, a subset of these altered proteins were mitochondrial genome encoded.

**Table 1:** Summary table of KEGG enrichment pathway analysis of heat stress related proteins in the control group

Pathway	ID	Protein name	Fold change^a^	Description
Oxidative phosphorylation				
	SPMIT.09	Atp8	0.41	F1-FO ATP synthase subunit 8
	SPMIT.01	Cox1	0.32	cytochrome c oxidase subunit 1
	SPMIT.11	Cox2	0.37	cytochrome c oxidase subunit 2
	SPMIT.04	Cox3	0.57	cytochrome c oxidase subunit 3
	SPAC1296.02	Cox4	0.66	cytochrome c oxidase subunit IV
	SPCC338.10c	Cox5	0.61	cytochrome c oxidase subunit V
	SPBC2F12.17	Cox7	0.29	cytochrome c oxidase subunit VII Cox7
	SPAC24C9.16c	Cox8	0.56	cytochrome c oxidase subunit VIII
	SPCC1442.08c	Cox12	0.67	cytochrome c oxidase protein VIb
	SPAC1071.10c	Pma1	0.49	plasma membrane P-type proton exporting ATPase, P3-type Pma1
	SPAC11E3.12	SPAC11E3.07	1.65	mitochondrial thioredoxin family protein, implicated in sulfur cluster assembly
	SPAC16E8.07c	Vph1	4.91	V-type ATPase V0 subunit a
	SPAC17A2.03c	Vma6	5.20	V-type ATPase V0 subunit d
	SPAC1B3.14	Vma3	3.94	V-type ATPase V0 subunit c
	SPAC343.05	Vma1	2.96	V-type ATPase V1 domain, subunit A
	SPAC637.05c	Vma2	3.56	V-type ATPase V1 subunit B
	SPAC11E3.07	Vma4	3.24	V-type ATPase V1 subunit E
	SPAC732.01	Vma11	3.38	V-type ATPase V0 subunit c'
Lysosome				
	SPAC16E8.07c	Vph1	4.91	V-type ATPase V0 subunit a
	SPAC17A2.03c	Vma6	5.20	V-type ATPase V0 subunit d
	SPAC19G12.10c	Cpy1	3.05	vacuolar carboxypep
	SPAC1B3.14	Vma3	3.94	V-type ATPase V0 subunit c (proteolipid subunit)
	SPAC732.01	Vma11	3.38	V-type ATPase V0 subunit c'
	SPCP1E11.06	Apl4	2.65	AP-1 adaptor complex gamma subunit Apl4
Phagosome				
	SPAC11E3.07	Vma4	3.24	V-type ATPase V1 subunit E
	SPAC16E8.07c	Vph1	4.91	V-type ATPase V0 subunit a
	SPAC17A2.03c	Vma6	5.20	V-type ATPase V0 subunit d
	SPAC1B3.14	Vma3	3.94	V-type ATPase V0 subunit c (proteolipid subunit)
	SPAC343.05	Vma1	2.96	V-type ATPase V1 domain, subunit A
	SPAC637.05c	Vma2	3.56	V-type ATPase V1 subunit B
	SPAC732.01	Vma11	3.38	V-type ATPase V0 subunit c'
Phospholipase D signaling pathway				
	SPAC2F7.16c	Pld1	0.63	phospholipase D, Pld1
	SPAC31G5.09c	Spk1	3.13	MAP kinase Spk1
	SPAC4A8.07c	Lcb4	0.41	sphingoid long chain base kinase
	SPBC1539.08	Arf6	0.32	ADP-ribosylation factor, Arf family Arf6
Tryptophan metabolism				
	SPAC6B12.04c	SPAC6B12.04c	0.46	2-aminoadipatetransaminase/kynurenine-oxoglutarate transaminase
	SPAC9E9.09c	Atd1	9.53	aldehyde dehydrogenase
	SPCC757.07c	Ctt1	0.63	catalase
Fatty acid biosynthesis				
	SPAC11G7.05c	Mct1	2.07	Mitochondrial [acyl-carrier protein] S-malonyltransferase Mct1
	SPAC26F1.04c	Etr1	2.15	enoyl-[acyl-carrier protein] reductase
	SPBC18H10.02	Lcf1	0.56	long-chain-fatty-acid-CoA ligase Lcf1

a Wild-type cells under heat-stress conditions/wild-type cells.

### Marked decrease in mitochondrial translation during heat stress

**Figure 2:**
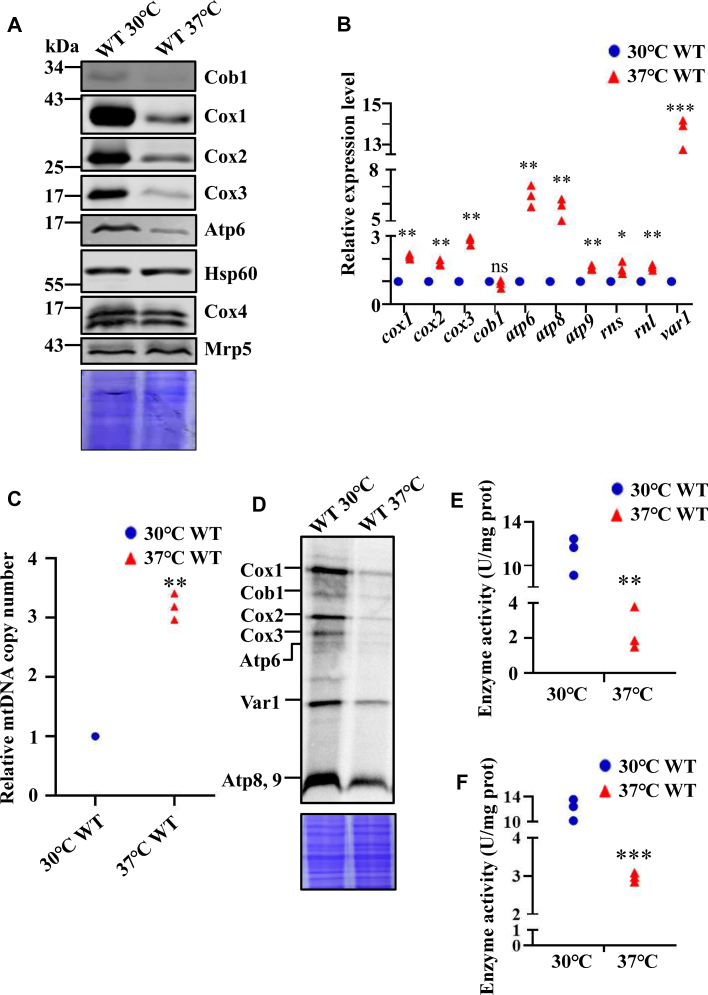
**Heat stress of WT impairs mitochondrial protein synthesis.***A*, heat stress affects the steady-state levels of mtDNA-encoded proteins. Mitochondrial extracts were prepared from WT cells under unstressed and heat stress conditions by spheroplast lysis, and analyzed by western blotting with anti-peptide Abs against mitochondrial-encoded Cob1, Cox1, Cox2, Cox3, Atp6, and Hsp60, as well as nuclear-encoded Cox4 and Mrp5. Coomassie Brilliant *Blue*-based total protein staining was adopted for loading control normalization. *B*, analysis of steady-state levels of mature mtRNAs and mitochondrial protein synthesis in WT cells under unstressed and heat stress conditions. The strains were cultured in YES medium for 12 h at 30 °C and 37 °C. Total RNA was isolated, and the mRNA expression levels were measured by qRT-PCR. The mRNA level of each gene was normalized to actin (*act1*) mRNA. Levels of mature mtRNAs in 30 °C WT cells were normalized and expressed as fold change over control 37 °C WT strain. Each dot represents an independent biological replicate. *C*, determination of mtDNA copy number in WT cells under 30 °C and 37 °C culture conditions by qPCR. Changes in mtDNA copy numbers were determined by measuring the relative mtDNA/nuclear DNA ratio by qPCR and expressed as relative mtDNA copy numbers normalized to that of the WT cells. Three independent experiments were performed. Error bars represent the SD of triplicates. (*D*) Heat stress significantly reduces the level of mitochondrial translation products of the WT strain. *In vivo* synthesis of mtDNA-encoded proteins in WT cells which cultured at 30 °Cand 37 °C. Mitochondrial translation products were labeled by incubating cells with [^35^S] methionine/cysteine for 3 h in the presence of anisomycin under heat stress conditions and unstressed conditions. Labeled proteins were separated by SDS-PAGE and analyzed by autoradiography. Coomassie staining of the gel (*bottom*) served as a loading control. The results presented are representative of multiple experiments. The enzyme activity of complex III (*E*) and complex IV (*F*) decreased in WT cells under heat shock conditions. Activity was measured in WT cells at 30 °C and 37 °C. Values are presented as means ± SD of three independent experiments. Statistical significance was determined by Student’s *t* test (∗*p* < 0.05, ∗∗*p* < 0.01, ∗∗∗*p* < 0.001).

We next analyzed the steady-state levels of mtDNA-encoded proteins under heat stress by western blotting. Analysis of mitochondrial protein extracts prepared from the unstressed and heat-stressed cells using specific antibodies revealed dramatic reductions in Cob1, Cox1, Cox2, Cox3, and Atp6 protein levels under heat stress (Fig. 2*A*). Future studies should determine whether this decrease derives from reduced mitochondrial RNA (mtRNAs) levels, altered mitochondrial DNA (mtDNA) copy number, or impaired mitochondrial protein synthesis.

To investigate whether heat stress affects mtRNA accumulation, we extracted total RNA from cells subjected to heat stress as well as from unstressed cells and performed qRT-PCR using primer pairs specific to these genes. Our findings indicate that the levels of almost all mRNAs and rRNAs were induced under heat stress (Fig. 2*B*).

To quantify potential changes in total mtDNA levels under heat stress, we used real-time quantitative PCR (qPCR) to determine the mtDNA to nuclear DNA ratios. We compared these ratios to those found in WT cells grown at 30 °C, following the methodology described in previous reports (25). Our results show an increase in mtDNA copy number under heat stress (Fig. 2*C*). These findings indicate that reduced levels of mitochondrial-encoded proteins occur independently of mtDNA or mRNA alterations. We consequently examined whether heat stress impairs mitochondrial translation, thereby reducing their steady-state levels.

We examined the effect of heat stress on mitochondrial protein synthesis. Mitochondrial translation products were labeled with [^35^S]-methionine/cysteine in unstressed and heat-stressed cells using anisomycin to inhibit cytosolic translation. Anisomycin was selected due to its low background signal under our conditions. Using the approach in *S. pombe*, we could identify all proteins synthesized by mitochondria based on their sizes and published results (26, 27, 28). As shown in Figure 2*D*, the levels of mtDNA-encoded proteins were substantially decreased in heat-stressed cells compared to unstressed cells, indicating that the synthesis of mtDNA-encoded proteins was downregulated under heat stress. We next assessed complex III and complex IV enzyme activities in WT cells grown at 30 °C and 37 °C to evaluate the impact of heat stress on mitochondrial respiratory chain function. Our results revealed decreases in both complex III and complex IV enzyme activities after heat stress in WT cells (Fig. 2, *E* and *F*).

### Heat stress impairs the association of mitochondrial translation initiation factors with the mt-SSU

**Figure 3:**
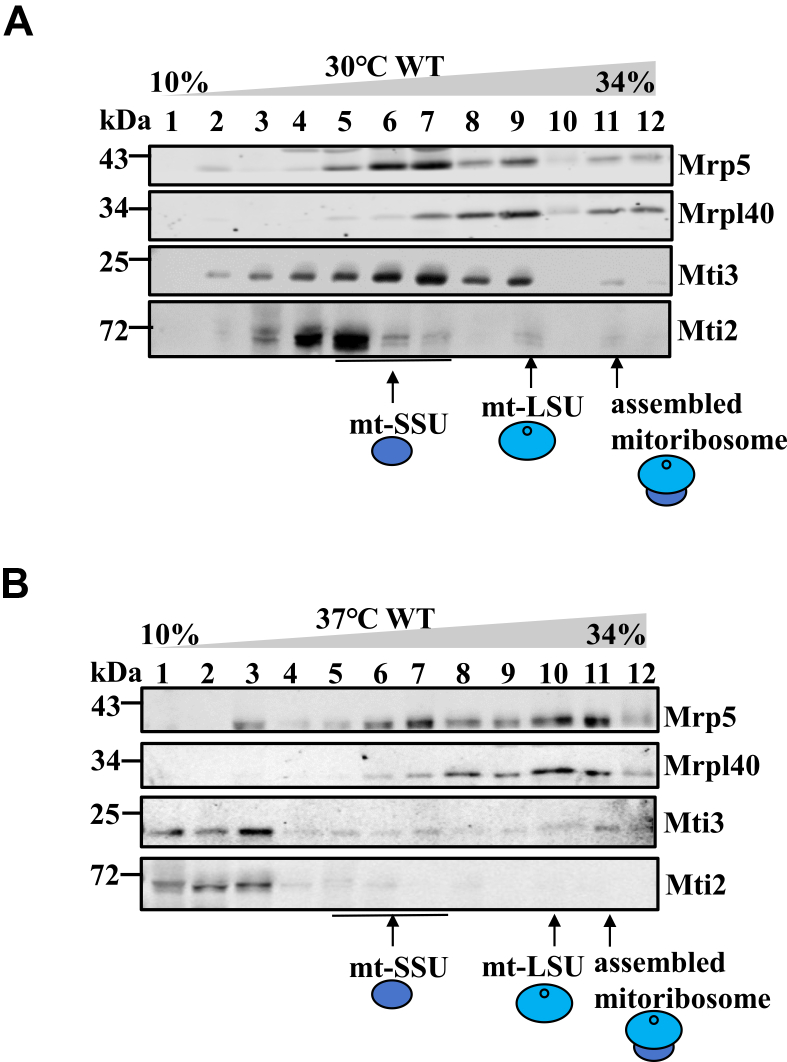
**Heat stress affects the association of the mitochondrial initiation factors with the mt-SSU.** Sucrose gradient analyses under unstressed (*A*), and heat stress (*B*) conditions. Mitochondrial extracts were prepared from spheroplasts and fractionated by sucrose gradient sedimentation. Proteins were TCA precipitated, separated by SDS/PAGE and analyzed by immunoblotting using abs against mitochondrial initiation factors Mti2 and Mti3, mitochondrial small ribosomal protein Mrp5, and mitochondrial large ribosomal proteins (Mrpl16 and Mrpl40). Arrows indicates fractions where the mitochondrial small ribosomal subunit (mt-SSU), the mitochondrial large ribosomal subunit (mt-LSU), and the assembled mitoribosome peak. M, molecular marker in kDa. C, total mitochondrial proteins.

Since heat stress could decrease mitochondrial translation, we assessed whether heat stress affects mitoribosome assembly using sucrose gradient sedimentation coupled with western blotting. Mitochondrial extracts from unstressed and heat-stressed cells were separated on sucrose gradients under conditions preserving intact mitoribosomes. The sedimentation profile of the small and large mitoribosomal subunits (mt-SSU/mt-LSU) and fully assembled mitoribosomes was determined by quantifying subunit protein levels (29). Heat stress did not impair the assembly of mitoribosomes or their subunits (Fig. 3, *A* and *B*). We next examined whether heat stress would affect the association of mitochondrial translation initiation factors (Mti2/Mti3) with mitoribosomes. Under control conditions, most of the Mti2 and Mti3 cosedimented with the mt-SSU, while a minor fraction associated with assembled mitoribosomes (Fig. 3*A*). In contrast, we found that heat stress caused dissociation of both factors from the mt-SSU and reduced their association with mitoribosomes (Fig. 3*B*).

### The mitochondrial translation can be restored by knocking out *ago1*

**Figure 4:**
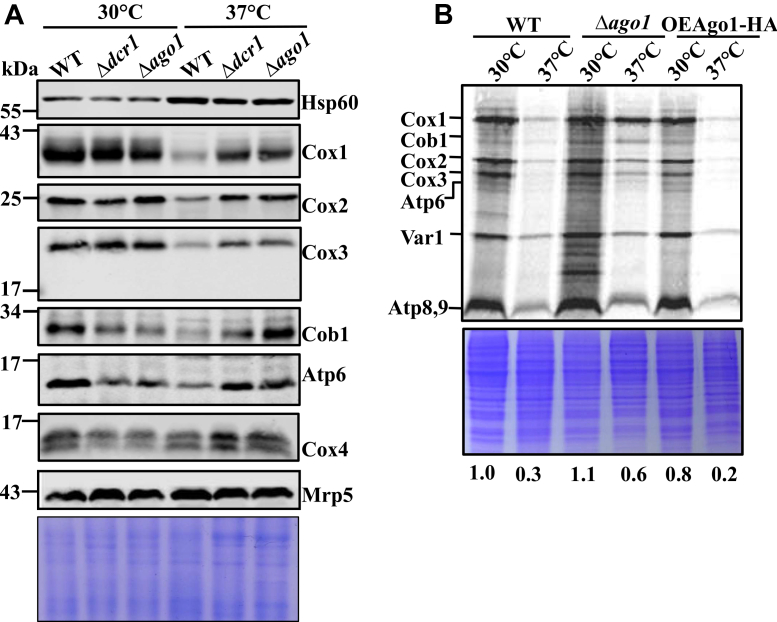
**Mitochondrial translation is restored by deletion of *ago1* under heat stress.***A*, analysis of steady-state levels of mitochondrial encoded protein in *ago1* and *dcr1* deletion cells under heat stress. Mitochondrial extracts were prepared from WT, *Δago1* and Δ*dcr1* cells under unstressed and heat stress conditions by spheroplast lysis and analyzed by western blotting with anti-peptide Abs against mitochondrial-encoded Cob1, Cox1, Cox2, Cox3, Atp6,and Hsp60, as well as nuclear-encoded Cox4, Mrp5, among them. Coomassie staining of the gel (*bottom*) served as a loading control. *B*, *in vivo* synthesis of mtDNA-encoded proteins in WT, *ago1* deletion (*Δago1*) and Ago1 overexpression (OEAgo1-HA) cells during unstressed or heat stress conditions. Deletion of *ago1* restored mitochondrial protein synthesis under heat stress. Mitochondrial translation products were labeled by incubating cells with [^35^S] methionine/cysteine for 3 h in the presence of anisomycin under heat stress conditions and unstressed conditions. Labeled proteins were analyzed by SDS-PAGE and autoradiography. Coomassie staining of the gel (*bottom*) served as a loading control. The results presented are representative of multiple experiments. The numbers below the bands denote the quantification of band intensity, taking Cox1 as a representative mitochondrial translation product. Signal intensities were normalized to the WT 30 °C control group.

Considering that nuclear-encoded factors regulate mitochondrial translation, we aimed to identify those involved in this process under heat stress. In the cytoplasm, AGOs facilitate miRNA-mRNA binding to inhibit translation. To examine the function of *ago1 or dcr1* in mitochondria, we generated deletion mutants of *ago1* and *dcr1* then assessed if their deletion affected mitochondrial protein levels in unstressed and heat-stressed cells. Deletion of *ago1* and *dcr1* restored steady-state protein levels of nearly all mitochondrial-encoded proteins reduced by heat stress (Fig. 4*A*). Moreover, reintroduction of *ago1* reversed this restoration, downregulating expression to wild-type levels under heat stress (Fig. S1). These observations demonstrate that Ago1 and Dcr1 mediate regulation of mitochondrial protein levels during heat stress.

To further explore the effect of *ago1* deletion on mitochondrial translation, we investigated the synthesis of mitochondrial proteins under heat stress. Mitochondrial translation products were pulse-labeled with [^35^S]-methionine/cysteine in stressed and unstressed cells using anisomycin to inhibit cytosolic translation. Comparison to unstressed cells, heat-stressed cells showed markedly decreased levels of mtDNA-encoded proteins (Fig. 4*B*). Moreover, protein translation was partially restored in *ago1-deleted* cells under heat stress (Fig. 4*B*). The results demonstrate that deletion of *ago1* restores translation levels of Cox1, Cox2, and Cox3 under heat stress (Figs. 4*B* and S2). Additionally, limited rescue effects are observed for Var1, Atp8, and Atp9, indicating complex-specific translational control mechanisms (Figs. 4*B* and S2). Conversely, Ago1 overexpression caused an additional reduction in protein translation levels (Fig. 4*B*). These observations suggest that Ago1 potentially plays a role in regulating the synthesis of mtDNA-encoded proteins under heat stress.

To determine whether reduced mitochondrial protein levels upon *ago1* knockout stem from impaired mtRNA transcription, we assessed mtRNA level by qRT-PCR in heat-stressed cells. This analysis showed there was no significant change compared to wild-type under heat stress (Fig. S3), indicating that Ago1 does not affect mtRNA transcription or stability in heat-stressed cells.

### Ago1 is involved in regulating translation though disrupting the mitochondrial translation initiation during temperature stress

**Figure 5:**
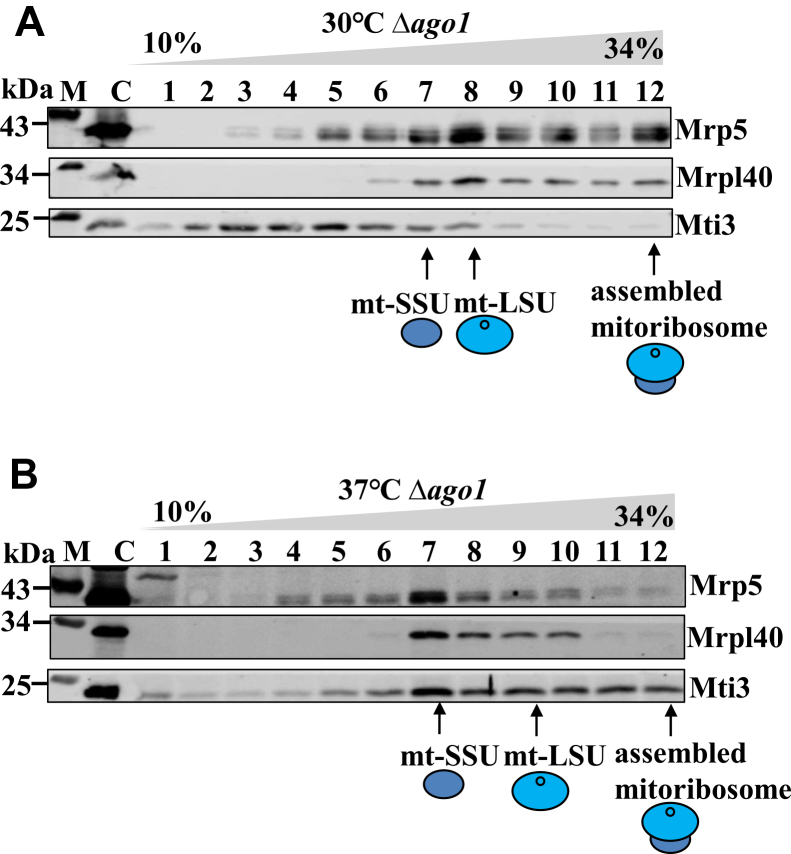
**Disruption of *ago1* affects mitochondrial translation.** Sucrose gradient analyses in Δ*ago1* cells under unstressed conditions (*A*) or heat stress conditions (*B*). Mitochondrial extracts were prepared from spheroplasts and fractionated by sucrose gradient sedimentation. Proteins were TCA precipitated, sceparated by SDS/PAGE and analyzed by Western blotting using antibodies against mitochondrial initiation factor Mti3, mitochondrial small ribosomal protein Mrp5, and mitochondrial large ribosomal proteins Mrpl40). Arrows indicate peak fractions of the mitochondrial small ribosomal subunit (mt-SSU), mitochondrial large ribosomal subunit (mt-LSU), and assembled mitoribosomes. M, molecular marker in kDa. C, total mitochondrial proteins.

To understand how *ago1* deletion restores mitochondrial-encoded protein level, we examined whether Ago1 is required for its association with the mt-SSU. Mitochondria isolated from *Δago1* cells under both unstressed and heat-stressed conditions were subjected to sucrose gradient centrifugation. Immunoblotting of gradient fractions revealed distributions of Mrp5, Mrpl40, and Mti3. *ago1* deletion rescued heat stress-induced dissociation of Mti3 from mitoribosomes (Fig. 5, *A* and *B*). These results indicate that Ago1 is involved in regulating translation by disrupting the mitochondrial translation initiation during temperature stress.

### Evidence for the presence and interaction of Ago1 with the mitochondria

**Figure 6:**
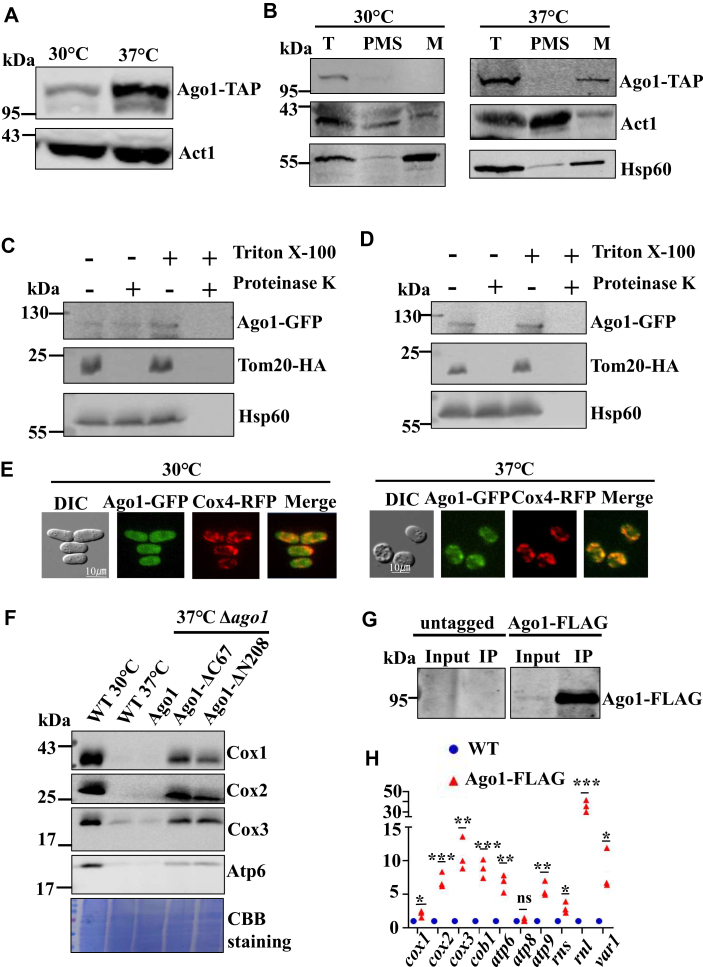
**Heat stress upregulates Ago1 protein levels and induced Ago1 to colocalize in the mitochondria in advance.***A*, heat stress induces Ago1 expression. Cells expressing chromosomally TAP-tagged Ago1 were grown at the indicated temperatures for 12 h, and the Ago1 protein levels were monitored by western blotting with anti-CBP Ab. Results are representative of three independent experiments. Actin detected by anti-Actin Ab served as a loading control. *B*, Ago1 localizes to mitochondria under heat stress. Mitochondria were isolated from cells expressing Ago1-TAP as described in Experimental procedures. Total cell extracts (T), mitochondria (M) and postmitochondrial supernatants (PMS) were analyzed by western blotting using anti-CBP Ab to detect Ago1-TAP, antibody against nuclear localized Actin (anti-Actin Ab),and the purified mitochondrial fraction (M) using the antibody against mtDNA-encoded Hsp60 (anti-Hsp60 Ab). *C*, Ago1 is located in the mitochondrial matrix. Isolated mitochondria were treated with proteinase K in the absence or presence of Triton X-100 as indicated. After precipitation with tricarboxylic acid, samples were analyzed by immunoblot analysis using anti-GFP, anti-HA and anti-Hsp60 Abs. Tom20-HA (outer mitochondrial membrane marker) and HSP60 (matrix marker) served as controls. The results are representative of three independent experiments. *D*, Ago1ΔC201-GFP is sensitive to proteinase K in intact mitochondria. Isolated mitochondria were treated with proteinase K in the absence or presence of Triton X-100 as indicated. After precipitation with tricarboxylic acid, samples were analyzed by immunoblot analysis using anti-GFP, anti-HA, and anti-HSP60 antibodies. Tom20-HA and HSP60 served as controls. *E*, Heat stress induces Ago1 localization into the mitochondria. Cells expressing Ago1-GFP and Cox4-RFP were grown at 30 °C or 37 °C. GFP signals were detected by fluorescence microscopy and photographed. Mitochondria was assessed by Cox4-RFP signals. All results were found to be reproducible in at least two experiments. *F*, analysis of steady-state levels of mitochondrial encoded protein in WT and Δ*ago1* mutant strains (*ago1*Δ [Ago1],*ago1*Δ [Ago1-ΔC67] (C-terminal 769–835aa truncated), *ago1*Δ [Ago1-ΔN208] (N-terminal 1–208aa truncated).) under unstressed (30 °C) and heat stress (37 °C) conditions, and analyzed by western blotting with anti-peptide antibodies against mtDNA-encoded Cox1, Cox2, Cox3 and Atp6. Coomassie brilliant blue (CBB) staining of the gel serves as a loading control. The results are representative of multiple independent experiments. *G*, Ago1 interacts with mt-mRNAs under heat stress. Mitochondrial extracts prepared from *S. pombe* strains expressing chromosomally FLAG-tagged Ago1 and untagged Ago1 (as a control) were immunoprecipitated with IgG agarose. *H*, total RNA was extracted from mitochondrial extracts (Input) and the IP and subjected to RT-PCR analysis with primers specific for mtDNA-encoded mRNAs as described above. Statistical significance was determined by the Student’s *t* test using the GraphPad Prism software (∗*p* < 0.05, ∗∗*p* < 0.01, ∗∗∗*p* < 0.001).

To investigate the role of Ago1 during heat stress in *S. pombe*, we examined the expression of Ago1 by Western blotting in the unstressed and heat-stressed cells. Ago1 increased during the exponential phase under heat stress (Fig. 6*A*). To test mitochondrial localization, mitochondria were isolated from cells expressing genomically tagged Ago1-TAP under its native promoters. Mitochondrial extracts were immunoblotted for Ago1-TAP, which revealed enrichment in heat-stressed fractions. As controls, the mitochondrial matrix protein Hsp60 was detected in the purified mitochondria (Fig. 6*B*). To confirm mitochondrial matrix localization of Ago1, we performed proteinase K assays. After protease K treatment to remove mitochondrial outer membrane-associated proteins and Triton X-100 permeabilization, Ago1 remained detectable in mitochondrial matrix fractions (Fig. 6*C*), demonstrating its stable localization in the matrix during exponential growth under heat stress. Additionally, in WT strain yAS56 expressing Ago1-GFP, Ago1 was cytoplasmic under normal conditions but mitochondrial in heat-stressed cells (Fig. 6*E*). Furthermore, truncation of the C-terminal (Δ769–834 aa) ablates mitochondrial matrix targeting (Fig. 6*D*). Crucially, complementation with this truncated variant failed to rescue temperature-induced suppression, establishing mitochondrial localization as essential for Ago1's regulatory function in mitochondrial protein expression (Fig. 6*F*). Our study also investigated the subcellular localization dynamics of Dcr1, a key component of the RNA interference complex. We isolated mitochondria from cells expressing Dcr1-GFP under its endogenous promoter and found its presence in the mitochondria-enriched fraction by Western blot analysis (Fig. S4*A*). The Dcr1-GFP-expressing yAS56 strain exhibited cytoplasmic localization but showed mitochondrial targeting in heat stress-dependent (Fig. S4*B*). These results imply that Ago1 and Dcr1 may be present in the mitochondria, but further research is required to determine their regulatory roles in mitochondrial function.

As established by Kwak *et al.* (30), the N-terminal domain of Ago1 facilitates small RNA loading. Accordingly, we generated an N-terminally truncated mutant (Ago1Δ1-208aa). While complementation of *Δago1* with full-length Ago1 restored mitochondrial protein expression to wild-type levels under heat stress, the truncated variant failed to rescue temperature-induced translational suppression (Fig. 6*F*), indicating that small RNA loading is essential for Ago1's regulatory function. The dependence on small RNA loading raised the possibility of direct Ago1-mtRNA engagement. We therefore probed physical interactions *via* RIP assays. To investigate potential physical interactions between Ago1 and mtRNAs, we performed RNA immunoprecipitation (RIP) assays. We first constructed a strain expressing a Flag tag integrated at the C-terminus of Ago1 at its own genomic locus (Ago1-Flag). This tag and additional tags described below did not apparently affect the *in vivo* function of the protein, as assayed by growth on rich medium (data not shown). In addition, all tagged proteins are of approximately the expected size. Ago1 was immunoprecipitated from mitochondrial extracts expressing Ago1-Flag using IgG agarose beads (Fig. 6*G*). RNA was purified from the total and immunoprecipitated fractions and analyzed by qRT-PCR using primers specific for mt-mRNAs. To distinguish between Ago1-Flag-associated RNA and RNA precipitated in a nonspecific way, untagged Ago1 cells were used in parallel. The resulting qRT-PCR products were sequenced to confirm the identities. qRT-PCR analysis identified that the association of mtRNAs with Ago1-Flag was significantly increased compared with untagged Ago1 (Fig. 6*H*). These results indicate that Ago1 may be associated with all mtDNA-encoded mRNAs.

## Discussion

Mitochondrial protein synthesis, which provides key components of the OXPHOS complexes is vital for mammals and *S. pombe*. In this study, we determined the impact of physiological heat stress on mitochondria in *S. pombe.* Proteomic analysis revealed a global view of heat stress impacts on mitochondria. We identified significant changes in mitochondrial pathways affected by heat stress, including oxidative phosphorylation, the V-type ATPase complex, proteolysis, and translation. These proteolytic and translational processes are functionally linked to energy metabolism demands, such as carbohydrate, fatty acid beta-oxidation, and the tricarboxylic acid cycle (31, 32). Given that mitochondria contain the highest protein density among organelles, oxidative phosphorylation and ATP binding represent core molecular functions. Protein hydrolysis and translation are essential for maintaining mitochondrial structure and function. The V-type ATPase complex delivers protons to maintain mitochondrial membrane potential. Notably, mtDNA-encoded proteins were significantly downregulated under heat stress. We found that while steady-state levels of mtDNA-encoded proteins were reduced under heat stress, there was no corresponding decrease in RNA levels. We further investigated the cause of this phenomenon and found that the protein downregulation was due to a decrease in mitochondrial translation.

After being subjected to thermal stress, the cytosolic compartment typically exhibits a highly coordinated response. Cytosolic translation is among the first processes to be downregulated, since it consumes a significant amount of energy, and the overall rate of protein synthesis decreases across the cell (33, 34, 35, 36). In addition, a specific subset of transcriptional units encoding heat shock proteins (HSPs) is activated, leading to increased synthesis of the corresponding proteins (18). However, thus far, little is known about the stress response of mtDNA-encoded proteins and their effects on the overall function of mitochondria under heat stress. Our research may lead to new insights into these functional aspects.

We showed that analysis of mitochondrial translation products of mtDNA-encoded proteins under heat stress reveals considerably impaired translation of almost all mtDNA-encoded proteins. However, qRT-PCR reveals that heat stress induces a mild increase in levels of mtDNA and most of mtRNAs to varying degrees. The reduced synthesis of mitochondrial proteins may serve to conserve cellular energy, as protein translation is a major energy-consuming process, accounting for up to 50% of cellular energy consumption depending on the organism (37, 38, 39). Additionally, the reduction in translation could prevent the synthesis of unnecessary proteins, allowing cells to better adapt to changing environmental conditions. Furthermore, as previously mentioned, the response to thermal stress also involves the activation of heat shock proteins, which serve as a typical stress response mechanism and offer cellular protection. Deletion of *ago1* specifically restored translation of Cox1, Cox2, and Cox3 under heat stress, but only partially rescued Var1, Atp8, and Atp9. This differential rescue pattern reveals complex-specific translational control. Furthermore, temperature-dependent suppression of mitochondrial translation likely requires coordinated regulation by multi-gene networks beyond Ago1. Overall, the decrease in mitochondrial protein synthesis may serve as a protective mechanism in response to environmental stress, helping to maintain normal cellular physiology. Despite the decrease in translation and steady-state levels of mitochondria-encoded OXPHOS polypeptides, we observed an increase in mitochondria-encoded RNAs. These observations may imply the existence of a compensatory mechanism aimed at counterbalancing the reduced production of proteins. Although we have shown that the translation of mtDNA-encoded proteins has decreased, it remains to determine how their translation is downregulated during heat stress.

Regulation of protein synthesis is crucial for controlling gene expression in response to various cellular cues, such as nutrient availability, stress response, and developmental signals. This regulation can occur at multiple stages of translation, including initiation, elongation, and termination. The initiation stage is where most of the regulation occurs, allowing for quick and reversible control of protein synthesis. In the cytoplasm, translational control primarily targets the initiation factors eIF2 and eIF4E. Impairment of these factors can affect a large portion of the translatome and elicit a rapid and robust reaction (40). Moreover, in mammalian cells, heat and proteotoxic stresses impact translation at the elongation stage, mainly through ribosome trapping or "5′-ribosome pausing" triggered by the altered cellular environment resulting from the accumulation of misfolded proteins (41, 42). The mitochondrial translation initiation factors interact cooperatively with the mitochondrial ribosomal small subunit (mt-SSU) during the initiation of mitochondrial protein synthesis. Our data revealed that heat stress affects the association of mitochondrial translation initiation factors Mti2 and Mti3 with the mt-SSU. This impairment directly underlies heat stress-induced mitochondrial translational suppression, which modulates protein production to maintain cellular viability.

Mitochondria are semi-autonomous organelles that carry their own small genome (mtDNA). They have their own translation machinery to generate the 13 protein subunits of the respiratory chain complexes encoded by the mitochondrial genome (43, 44). Since most of the proteins that make up the mitochondrial translation machinery are encoded by nuclear genes, synthesized in the cytoplasm, and then targeted to the mitochondrial compartment, mitochondrial and cytoplasmic translation are regulated by nuclear-encoded factors in order to coordinate the timely synthesis of OXPHOS complexes (44, 45). We are interested in identifying the nuclear-encoded proteins that are involved in regulating the translation of mitochondrial-encoded proteins under heat stress. Given the established role of AGOs in facilitating miRNA-mRNA interactions to suppress cytoplasmic translation (46), we propose to investigate whether analogous mechanisms regulate mitochondrial translational adaptation during heat stress. Human AGO2 has been identified in mitochondria and shown to co-immunoprecipitate with mitochondrial tRNA^Met^ (47, 48). Moreover, several studies report AGO2 localization to subcellular compartments, suggesting its potential role in maintaining organellar function (47, 49, 50, 51, 52). Notably, *S. pombe* contains Ago1, a homolog of human AGO2, which functionally compensates for human AGO2 (53, 54, 55, 56). Similarly, Dcr1, the *S. pombe* homolog of human Dicer, is also present. Through bioinformatics analysis, we have further identified a mitochondrial target signal for Dcr1 in *S. pombe* (57, 58). As core components of the RNA interference pathway, Ago1 and Dcr1 are both necessary for transcriptional and post-transcriptional silencing in fission yeast. In our studies, we observed that deletion of *ago1* can partially restore the translation and steady-state levels of mitochondrial encoded proteins, while knocking out *dcr1* also recovers mitochondrial-encoded protein steady-state levels. To our knowledge, this is the first report showing that Ago1 is required for the regulation of mitochondrial translation initiation under heat stress. Furthermore, our findings also demonstrating mitochondrial localization of Ago1. Under heat stress, Ago1 accumulates in the mitochondrial matrix. C-terminal truncation (Δ769–834aa) abolishes this localization and fails to rescue temperature-induced translational suppression, confirming that mitochondrial localization is essential for Ago1's regulatory function in mitochondrial protein expression. Moreover, we reveal that heat-induced translational suppression requires Ago1's N-terminal domain (responsible for small RNA loading) and that Ago1 physically interacts with mitochondrial RNAs (mtRNAs), implicating potential RNA interference (RNAi) activity within mitochondria. While these findings suggest mitochondrial RNAi activity, the mechanistic basis for this phenomenon requires elucidation. We hypothesize two possible explanations for this phenomenon. The first possibility proposes that core components of the RNA interference pathway (Ago1 and Dcr1) likely participate in mitochondrial RNA interference within the mitochondria, which can directly impact the translation of mitochondrial-encoded proteins. Given the recent observations that various miRNAs were detected in isolated mitochondria under heat stress, we reasoned that reduced translation in the mitochondria might account for increased miRNA targeting to mitochondria under heat stress. Alternatively, it is possible that Ago1 and Dcr1 regulate the expression of translation-related genes in the cytoplasm through RNAi, which subsequently affects the translation of mitochondrial-encoded proteins. Further research is necessary to fully understand the relationship between RNA interference components and mitochondrial-encoded protein translation.

**Figure 7:**
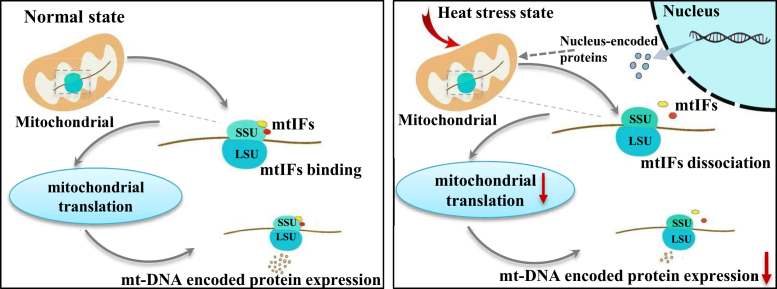
**Schematic summary model.** Heat stress disrupts the association of mitochondrial initiation factors with mitoribosomes, leading to reduced translation of mtDNA-encoded transcripts and decreased mtDNA-encoded protein levels. Nuclear-encoded proteins, such as Ago1, modulate this interaction, thereby regulating mtDNA-encoded protein translation.

Our study reveals that heat stress disrupts the interaction between mitochondrial translation initiation factors and mitoribosomes, leading to reduced translation of mtDNA-encoded transcripts and consequently decreased levels of mtDNA-encoded proteins. Notably, nuclear-encoded proteins like Ago1 play a regulatory role by modulating the association of mitochondrial translation initiation factors with mitoribosomes, thereby influencing the translation of mtDNA-encoded proteins. Collectively, these results underscore the importance of mitochondrial translational regulation as a key adaptive mechanism in response to heat stress, providing novel insights into how cells maintain protein homeostasis and survival under heat stress (Figure 7).

## Experimental procedures

### Strains and media

*S. pombe* strains used in this study are listed in Table S1. Strains deleted for *ago1* or *dcr1* were generated by the one-step gene replacement method (59). Strains carrying epitope-tagged genes were created by homologous recombination at the C-termini of the genes. Plasmid pJK148 was used to introduce the green fluorescent protein (GFP) tag into *ago1* or *dcr1*. The full-length *ago1* and mutant fragments (the N-terminal truncated 624 bp, and the C-terminal truncated 201 bp) were all cloned into the plasmid vector pYJ19, which is driven by the highly expressed promoter *nmt41* and carries a GFP tag. Strains overexpressing Ago1 tagged with c-GFP (Ago1-GFP) or Dcr1 tagged with c-GFP (Dcr1-GFP) under the control of the *nmt1* promoter were constructed as previously described (25, 60). Strains expressing Ago1 tagged with the TAP tag (Ago1-TAP) from its endogenous promoter and native genomic locus were also constructed as described (25, 60). To construct the Ago1 overexpression strain, the *tif51* promoter with the *hphMX6* marker was utilized. All epitope tags were added at the C-termini, and all epitope-tagged strains were verified by PCR, DNA sequencing and Western blot analysis. Strains expressing Cox4 tagged with c-RFP (Cox4-RFP) were constructed similarly.

### Cell growth assay

For heat stress condition, cells were grown in (YES) medium with the appropriate supplements (0.5% yeast extract, 3% (w/v) glucose, and 225 mg/l each adenine, histidine, leucine, and uracil) or 3% glycerol and 0.1% glucose for respiratory growth (61, 62), then they were shifted to 37 °C and cultured for an additional 12 h before harvesting. For unstressed conditions, yeast cells were grown in YES overnight. For heat stress condition, cells were performed by growing cells in YES medium at 30 °C for 3 h, then cells were shifted to 37 °C and continued to grow for 12 h before harvesting. Samples were collected at the indicated time points (in h), normalized to an OD_600_ of 0.2, serially diluted 5-fold, and spotted on solid rich medium. The cultures were photographed after two or 4 days of incubation at 30 °C or 37 °C.

### Proteomic analysis

Quantitative proteomics using iTRAQ labeling and liquid chromatography electrospray ionization tandem mass spectrometry (LC-MS/MS) analysis Proteomic analysis was performed to identify proteins differentially expressed between 4 μg/ml miltefosine-treated cells and untreated cells. Mitochondrial protein extracts were prepared, and freshly isolated mitochondria (∼2 mg of mitochondrial proteins) were added on top of 4 ml sucrose and ultracentrifugated at 260,800*g* for 3 h at 4 °C in a SW 60Ti centrifuge (Beckman Coulter). The refined mitochondrial proteins were sent to Shanghai Personalbio Technology Co, Ltd, for iTRAQ coupled with LC-MS/MS analysis. ITRAQ tags 114, 115, 116, and 117 (AB Sciex) were used to label each sample containing 100 μg peptides following the manufacturer’s instructions. The labeled peptides were fractionated and dried for LC-MS/MS analysis. Then heat stress proteins were identified by an Easy-nLC binary buffer system coupled with a Q-Exactive mass spectrometer (Thermo Fisher Scientific). Proteins with a statistically significant label ratio of more than 1.2 and *p*-value < 0.05 were considered to be differentially expressed proteins (DEPs). Yeast samples from two biological replicates were used. Raw data can be accessed through ProteomeXchange database accession number PXD060104.

### Quantitative real-time RT-PCR

RNA was isolated from *S. pombe* cells under unstressed or heat stress conditions using the E.Z.N.A. Yeast RNA Kit (OMEGA BIO-TEK). Contaminating genomic DNA in RNA samples was removed by treatment with RNase-free DNase (Fermentas). RNA samples were reversed transcribed with the oligo (dT)_16_ primer using HiScript III RT SuperMix for qPCR Kit (Vazyme). Comparative qPCR analysis was performed using the StepOne RT-PCR system (Applied Biosystems) with each primer set (Table S2). All reactions were run in triplicate. Data analysis was performed by StepOne Software. The fold change in gene expression was calculated using the 2^−ΔΔCt^ method. The C_t_ values were normalized against that of actin (*act1*) mRNA from the same preparations to give the ΔC_t_ values. The ΔΔC_t_values were calculated by subtracting the normalized ΔC_t_ value of the wild-type cells from the normalized ΔC_t_ value of cells.

### Western blot analysis

*S. pombe* whole-cell extracts were prepared by alkaline extraction (63) or by breaking cells with glass beads using a FastPre-24 bead beater (MP Biomedicals) (64). Mitochondrial protein extracts were prepared as described above (10). Proteins were resolved by electrophoresis on SDS-PAGE, and the separated protein bands were transferred electrophoretically to the nitrocellulose membranes (GE Healthcare). Blots were probed with anti-Cox1 (1:1000), anti-Cox2 (1:1000), anti-Cox3 (1:400), anti-Cox4 (1:1000) and anti-Cob1 (1:500), anti-Atp6 (1:400), anti-Mrp5 (1:5000), anti-Hsp60 (1:1000; Sangon Biotech), anti-FLAG (1:1000; Sigma), anti-CBP (1:1000; GenScript), and anti-GFP (1:5000; Sigma-Aldrich) Abs. Secondary Abs used were IRDye 800CW conjugated goat anti-rabbit or anti-mouse Abs (LI-COR Biosciences). Bands were detected using an Odyssey near-infrared fluorescence scanner (LI-COR Biosciences).

### Pulse-labeling of mitochondrial translation products

*In vivo* labeling of mitochondrial translation products was performed according to previous study (25). Basically, WT, Δ*ago1* and Ago1*-overexpressing* cells were cultured to exponential phase in complete medium containing 0.1% glucose and 5% raffinose. A total of 1.5 OD_600_ of yeast cells were collected and suspended in 500 μl of reaction buffer containing 1 mg/ml anisomycin. After 15 min of preincubation at 30 °C, 8 μl of [^35^S]-methionine/cysteine mix (NEG-072; PerkinElmer Life Science) was added and the cell suspension were incubated at 30 °C for 3 h. After labeling, cells were pelleted by centrifugation at 10,000*g* for 1 min. The cell pellet was then suspended in 75 μl solubilization buffer (1.8 M NaOH, 1 M *β*-mercaptoethanol, 10 mM PMSF) and 500 μl of H_2_O was added to dilute the cell solubilization buffer. Proteins were precipitated by the addition of an equal volume of 50% trichloroacetic acid. The precipitates were washed, solubilized in SDS-PAGE loading buffer, and then separated by SDS-PAGE. The proteins were blotted onto nitrocellulose membranes and signals were detected by using the Cyclone Plus Storage Phosphor System (PerkinElmer).

### Fluorescence microscopy

Overnight cultures of strains expressing GFP-tagged Ago1, GFP-tagged Dcr1 or RFP-tagged Cox4 were diluted to an OD_600_ of 0.2 and grown for the designated time points. The green and red fluorescence signals were captured using a Zeiss Axio Imager A1 microscope (Zeiss) with excitation wavelengths of 488 nm and 579 nm, respectively. Images were obtained on a Zeiss Axio Imager A1 microscope (Zeiss) equipped with a PCO Sensicam CCD-camera (PCO Sensicam, Kelheim, Germany) and data were processed using MetaMorph image processing software (Universal Imaging).

### Sucrose density gradient analysis

Freshly isolated mitochondria (∼2 mg of mitochondrial proteins) were incubated in the lysis buffer (20 mM HEPES, pH 7.4, 100 mM KCl, 10 mM MgCl_2_, 0.5 mM PMSF, EDTA-free complete protease inhibitor (Roche), and 1% DG). The lysate was centrifuged, and the supernatant was layered on top of the 10 to 34% sucrose gradient containing 20 mM HEPES, pH 7.4, 100 mM KCl, 10 mM MgCl_2_, 0.5 mM PMSF, EDTA-free complete protease inhibitor (Roche Applied Science) and 0.1% DG, followed by ultracentrifugation. 12 fractions were collected and proteins were precipitated by adding 50% (w/v) trichloroacetic acid (TCA) overnight at −20 °C. Proteins were resolved on 12% SDS/PAGE and analyzed by immunoblotting with anti-Mrp5, anti-Mrpl40, anti-Mti2 Abs and anti-Mti3 Abs.

### Determination of mtDNA copy number

The mtDNA copy number was determined as previously described. Briefly, yeast cells were inoculated into YES and cultured overnight at 30 °C or 37 °C. Cells were diluted into fresh YES medium to an OD_600_ of 0.2 and then harvested for gDNA extraction in the logarithmic growth phase (OD_600_ of 4 ∼ 6). The qPCR was performed using KAPA HiFi HotStart Uracil + ReadyMix (Roche, Pleasanton, CA, USA) following the manufacturer’s instructions. The median of Ct values for mtDNA-encoded genes (*cob1*, *cox1*, *cox3*, and *atp9*) and nuclear DNA-encoded genes (*spo12*, *ace2*, and *exg1*) were used to estimate mtDNA and nuclear DNA levels, respectively. The fold change in the mtDNA copy number of the mutant relative to the WT strain (set to 1) was calculated using Eq.2^−ΔΔCt^.

### RNA immunoprecipitation

RNA immunoprecipitation (RIP) was performed as previously described (65). WT cells expressing chromosomally encoded FLAG-tagged Ago1 or untagged Ago1 (yAS56) were grown in YES medium and harvested after incubation at 37 °C for 12 h. Mitochondria were isolated as described above (10). Mitochondria were lysed in lysis buffer [20 mM Tris-HCl, pH 7.4, 100 mM NaCl, 0.7% n-dodecyl *β*-D-maltoside (DDM), and 200 U RNaseOUT (Invitrogen) and protease inhibitors]. The mitochondrial extracts were diluted 1:2 with lysis buffer without DDM and incubated with 20 μl of IgG agarose beads for 4 h at 4 °C with constant rotation. Following extensive washing with lysis buffer, bound RNAs were eluted by incubating the beads in tobacco etch virus protease cleavage buffer (10 mM Tris-HCl,pH 8.0, 300 mM NaCl, 0.1% Igepal CA-630, 200 U/ml RNaseOUT, 170 U/ml tobacco etch virus,and protease inhibitors). Both total RNA and immunoprecipitated RNA were extracted using phenol and purified with the miRNeasy Micro Kit (Omega). Purified RNAs were reverse-transcribed with HiScript III RT SuperMix for qPCR (+gDNA wiper) (Vazyme). The cDNA was used as the template to using Taq Pro Universal SYBR qPCR Master Mix (Vazyme) and primers (Table S2) targeting the 5′-UTRs of mtDNA-encoded genes. It should be noted that RT-PCR was quantitative under the conditions used here.

### Enzyme activity assay

*S. pombe* cells were inoculated into YES and cultured overnight at 30 °C or 37 °C. Cells were diluted into fresh YES medium to an OD_600_ of 0.2. Whole-cell extracts were obtained by breaking cells with glass beads using a FastPre-24 bead beater (MP Biomedicals) (64). Complex III (CoQ-cytochrome c reductase) and Complex IV (Cytochrome c oxidase) activity were determined using mitochondrial respiration complex III activity assay kit (Solarbio Science & Technology Co, Ltd) and mitochondrial respiration complex III activity assay kit (Solarbio Science & Technology Co, Ltd) following the manufacturer’s instructions.

### Protease K protection assay

Intact mitochondria were isolated from *S. pombe* cells and divided into four aliquots. One aliquot was left untreated, a second was incubated with 1% Triton X-100, a third was incubated with 50 μg/ml Proteinase K on ice for 30 min, and the fourth was pretreated with 1% Triton X-100 before Proteinase K digestion. Reactions were stopped with 2 mM PMSF. Samples were resolved by SDS-PAGE and analyzed by Western blotting. Matrix-localized Ago1 and Hsp60 were protected unless membranes were permeabilized, whereas the outer membrane marker Tom20 was degraded by Proteinase K alone.

## Data availability

The mass spectrometry proteomics data can be accessed through ProteomeXchange database accession number PXD060104. All data that support the findings of this study are contained within the article and its supporting information.

## Supporting information

This article contains supporting information.

## Conflict of interest

The authors declare the following financial interests/personal relationships which may be considered as potential competing interests: The authors declare that they have no conflicts of interest with the contents of this article.
