# Supplementary material for: Ago1 is required for the regulation of mitochondrial translation under heat stress in Schizosaccharomyces pombe
Source: J Biol Chem. 2026 Jun 4;302(7):113235. doi: 10.1016/j.jbc.2026.113235 (PMC13324456; doi:10.1016/j.jbc.2026.113235)
Supplement: Tables S1-S2 [file mmc1.docx]

Table S1. List of *S. pomb*e strains used in this study

| Strain | Genotype | Source |
| --- | --- | --- |
| yAS56 | *h^+^ leu1-32 ura4-D18* | Lab stock |
| Δ*ago1* | *h^+^ leu1-32 ura4-D18 Δago1::kanMX6* | This study |
| Δ*dcr1* | *h^+^ leu1-32 ura4-D18 Δdcr1::kanMX6* | This study |
| Ago1-TAP | *h+ leu1-32 ura4-D18 ago1::[ago1-TAP-hphMX6]* | This study |
| Tom20-HA | *h+ leu1-32 ura4-D18 tom20::[tom20-HA-kanMX6]* |  |
| Dcr1-GFP | *h^+^ leu1-32 ura4-D18 dcr1::[dcr1-GFP-hphMX6]* | This study |
| Cox4-RFP | *h^+^ leu1-32 ura4-D18 cox14::[cox4-RFP-kanMX6]* | This study |
| pJK148-Ago1-GFP | *h^+^ leu1-32 ura4-D18 ago1::[ago1-nmt41-GFP-leu1^+^]/ pJK148* | This study |
| pJK148-Dcr1-GFP | *h+leu1-32 ura4-D18 dcr1::[dcr1-nmt41-GFP-leu1^+^]/ pJK148* | This study |
| pJK148-Ago1-GFP-Cox4-RFP | *h+leu1-32 ura4-D18 ago1::[ago1-nmt41-GFP-leu1^+^]/ pJK148 cox4::[cox4-RFP-kanMX6]* | This study |
| pJK148-Dcr1-GFP-Cox4-RFP | *h+leu1-32 ura4-D18 dcr1::[dcr1-nmt41-GFP-leu1^+^]/ pJK148 cox4::[cox4-RFP-kanMX6]* | This study |
| OEAgo1-HA | *h^+^ leu1-32 ura4-D18 ago1::[ago1-tif51-3HA-hphMX6]/pTif51* | This study |
| pYJ19-Ago1-GFP | *h^+^ leu1-32 ura4-D18 ago1::[ago1-nmt41-GFP-leu1^+^]/ pYJ19* |  |
| pYJ19-Ago1-ΔN208-GFP | *h+ leu1-32 ura4-D18 ago1::[ago1ΔN208-nmt41-GFP-leu1+]/ pYJ19* |  |
| pYJ19-Ago1-ΔC67-GFP | *h+ leu1-32 ura4-D18 ago1::[ago1ΔC67-nmt41-GFP-leu1+]/ pYJ19* |  |
| pYJ19-Ago1-GFP-Δ*ago1* | *h^+^ leu1-32 ura4-D18 ago1::[ago1-nmt41-GFP-leu1^+^]/ pYJ19 Δago1::kanMX6* |  |
| pYJ19-Ago1-GFP-Tom20-HA | *h^+^ leu1-32 ura4-D18 ago1::[ago1-nmt41-GFP-leu1^+^]/ pYJ19 tom20::[tom20-HA-kanMX6]* |  |
| pYJ19-Ago1-ΔN208-GFP-Δ*ago1* | *h+ leu1-32 ura4-D18 ago1::[ago1ΔN208-nmt41-GFP-leu1+]/ pYJ19 Δago1::kanMX6* |  |
| pYJ19-Ago1-ΔN208-GFP-Tom20-HA | *h+ leu1-32 ura4-D18 ago1::[ago1ΔN208-nmt41-GFP-leu1+]/ pYJ19 tom20::[tom20-HA-kanMX6]* |  |
| pYJ19-Ago1-ΔC67-GFP-Δ*ago1* | *h+ leu1-32 ura4-D18 ago1::[ago1ΔC67-nmt41-GFP-leu1+]/ pYJ19 Δago1::kanMX6* |  |
| pYJ19-Ago1-ΔC67-GFP-Tom20-HA | *h+ leu1-32 ura4-D18 ago1::[ago1ΔC67-nmt41-GFP-leu1+]/ pYJ19 tom20::[tom20-HA-kanMX6]* |  |

Table S2. List of primers used in this study

| Gene | Primer (5’ to 3’) |
| --- | --- |
| Prime for deletion of *ago1* | |
| Δ*ago1*-up-F | GAACCTCCCGGTATTGTTAATATAG |
| Δ*ago1*-up-R | GTATTCTGGGCCTCCATGTCCCTACTCACTCTTGAATCATAAAGCTT |
| Δ*ago1*-kanMX6-F | TATGATTCAGAGTGAGTAGGGACATGGAGGCCCAGAATACCTTAT |
| Δ*ago1*-kanMX6-R | TGCATGCAATCCATCAAACACAGTATAGCGACCAGCATTC |
| Δ*ago1*-down-F | GAATGCTGGTCGCTATACTGTGTTTGATGGATTGCATGCAATAAG |
| Δ*ago1*-down-R | CTTAACACAGTATGCAATGTTTG |
|  |  |
| Primer for verification of *ago1* deletion | |
| Δ*ago1*-yz-up-F | GGTAAGCTGTTTAACGAG |
| Δ*ago1*-yz-hphMX6-R | CAGTCCCGGCTCCGGATCGG |
| Δ*ago1*-yz-hphMX6-F | CCGATCCGGAGCCGGGACTG |
| Δ*ago1*-yz-down-R | GATGTATGATGAAGGCAGC |
|  |  |
| Prime for deletion of *dcr1* | |
| Δ*dcr1*-up-F | ACCGAATCATTCTAGCATAAAG |
| Δ*dcr1*-up-R | GTATTCTGGGCCTCCATGTCTCAAAAAGAAAATAAAGGCG |
| Δ*dcr1*-kanMX6-F | CGCCTTTATTTTCTTTTTGAGACATGGAGGCCCAGAATAC |
| Δ*dcr1*-kanMX6-R | GAAATACTGTATATTTCAAGTCCAGTATAGCGACCAGCATTCAC |
| Δ*dcr1*-down-F | GTGAATGCTGGTCGCTATACTGGACTTGAAATATACAGTATTTC |
| Δ*dcr1*-down-R | GCCGAATTACTAGATTCATCC |
|  |  |
| Primer for verification of dcr*1* deletion | |
| Δ*dcr1*-yz-up-F | CATTAAGTGGATACTAATTAGCAAC |
| Δ*dcr1*-yz-hphMX6-R | CAGTCCCGGCTCCGGATCGG |
| Δ*dcr1*-yz-hphMX6-F | CCGATCCGGAGCCGGGACTG |
| Δ*dcr1*-yz-down-R | GGTATCTAATTCATGATCAAAACTC |
|  |  |
| Primer for strain expressing Ago1-TAP | |
| Ago1-TAP-up-F | CGATGATACTTTTGTTGAGACATCAGAAGCATCAATGGATCAAGAAGTAAAGCCGCTTTTAGCTCTATCAAGTAAATTGAAAACAAAGATGTGGTATATGCGGATCCCCGGGTTAATTAA |
| Ago1-TAP-up-R | AAAAACAGAAGCAGATTTAATAAGGAAGTAAAAGTTGTGGGCAATCCAGTAGTCAATCGTATATCTATTTCATTACTTATTGCATGCAATCCATCAAACAGAATTCGAGCTCGTTTAAAC |
|  | |
| Primer for strain expressing pJK148-Ago1-GFP | |
| Ago1-PstⅠ-F | AAAACTGCAGATGTCGTATAAACCAAGCTC |
| Agox1-SalⅠ-R | ACGCGTCGACCATATACCACATCTTTGTTTTCAA |
|  |  |
| Primer for strain expressing pJK148-Dcr1-GFP | |
| Dcr1-PstⅠ-F | GGAATTCGATATCAAGCTTATCATGGATATTTCAAGTTTTCTACTTCC |
| Dcr1-SalⅠ-R | TGCAGCCCGGGGGATCCACAGTCAAACTTTTTAACTTTTCCATC |
|  |  |
| Primer for strain expressing OEAgo1-HA | |
| Ago1-5’-F | GCAACAACAACAGAACTCGAGATGTCGTATAAACCAAGCTCAGAAATAGCT |
| Ago1-3’-R | TAAAAGATGTTAATTAACCCGGGTTACATATACCACATCTTTGTTTTCAATTTACTTGATAGAGCTAAAAG |
| Primer for strain expressing pYJ19-Ago1 truncated plasmid | |
| nmt41-ago1-f | CTTATAGTCGCTTTGTTAAATGTCGTATAAACCAAGC |
| nmt41-ago1-rago1-GFP-fago1-GFP-rnmt41-ago1ΔN208-fnmt41-ago1ΔN208-rnmt41-ago1ΔC201-fnmt41-ago1ΔC201-r | GCTTGGTTTATACGACATTTAACAAAGCGACTATAAGCAAAGATGTGGTATATGAGTAAAGGAGAAGAACGTTCTTCTCCTTTACTCATATACCACATCTTTGCTTATAGTCGCTTTGTTAATCTTTACTTCAGATCCGGATCTGAAGTAAAGATTAACAAAGCGACTATAAGCACTGTGTTATAATCTTAGTAAAGGAGAAGAACGTTCTTCTCCTTTACTAAGATTATAACACAGTG |
| Primers for qRT-PCR | |
| actin-RT-F | TCCGCTCTTAACATCTCATGAGG |
| actin-RT-R | AAGGCTAGCTCTGCATTCGTCTAT |
| cob1-RT-F | GCCTTTTGTTATTGCTGCTTTA |
| cob1-RT-R | GTTATCAAATCTTTTATCAGATAAT |
| cox1-RT-F | TGGACGGTATATCCACCACT |
| cox1-RT-R | GTCGCTATTAAATTTACTGATCC |
| cox2-RT-F | AAGTGGTGATGTTATCCATAGTTGG |
| cox2-RT-R | AGATACACCTTGAACAACAATAGGC |
| cox3-RT-F | CCACCAGTAGGAATAGCAGATAAAA |
| cox3-RT-R | TGAGCATAAGTTAAACTAGCACCAG |
| atp6-RT-F | TACCTTCTGGTACTCCTACTCC |
| atp6-RT-R | TAGCACCTAATCGAATACCTAAACTT |
| atp8-RT-F | ATGCCACAATTAGTACCATTCT |
| atp8-RT-R | AAAGAACTTATAATAGATCTTGAG |
| atp9-RT-F | GGTGCTGGTGTTGGTATTGGA |
| atp9-RT-R | ACCTGTAGCTTCTGTTAAGGCG |
| rns-RT-F | GAAGGAGGAATTGCGAGTAATCAC |
| rns-RT-R | CGACTTAACACTAATTGCACAACACC |
| var1-RT-F | AGAGCTCTTCCTATTTCAACTCCTT |
| var1-RT-R | ACCTTTCCATCCTTTTGGTACA |
|  |  |
| Primers for determination of mtDNA copy number | |
| spo12-F | TCGGCTCTAAGAAGGTATCTGTATC |
| spo12-R | AGTACCAGATCTGCCTGAGTAGTTG |
| ace2-F | CAAGACAAAATCTACTCCAAGTCGT |
| ace2-R | CATTAACCAAGTAGCGAGAACGTAT |
| exg1-F | CTACCTCGGTTAATTGGACTTTGTA |
| exg1-R | TCTTCAGGATCCTACATAGAAAACC |
| cox1-F | CGGTGTTGTTAGTCACATTATTCCT |
| cox1-R | TGCAGCACTGAAATAAGCTCTAGTA |
| cox3-F | CCACCAGTAGGAATAGCAGATAAAA |
| cox3-R | TGAGCATAAGTTAAACTAGCACCAG |
| cob1-F | TGCAAATGGTGCTAGTTTCTTC |
| cob1-R | TAGTAATAACAGTTGCACCCCAGA |
| atp9-F | GGTGCTGGTGTTGGTATTGGA |
| atp9-R | ACCTGTAGCTTCTGTTAAGGCG |
