# Supplementary figures and images for: Ago1 is required for the regulation of mitochondrial translation under heat stress in Schizosaccharomyces pombe
Source: J Biol Chem. 2026 Jun 4;302(7):113235. doi: 10.1016/j.jbc.2026.113235 (PMC13324456; doi:10.1016/j.jbc.2026.113235)

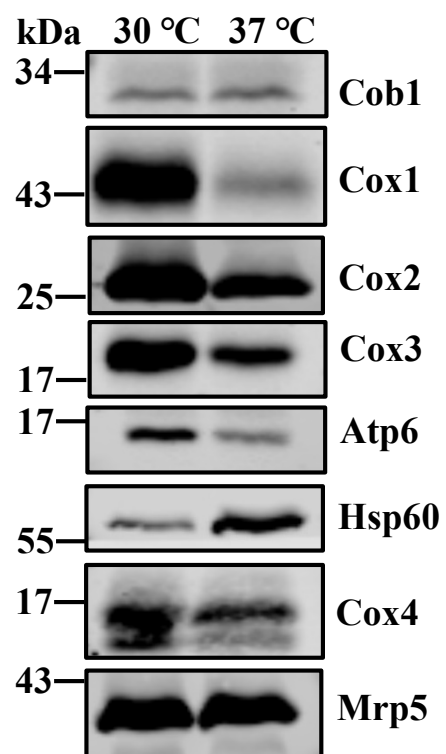

Fig. S1

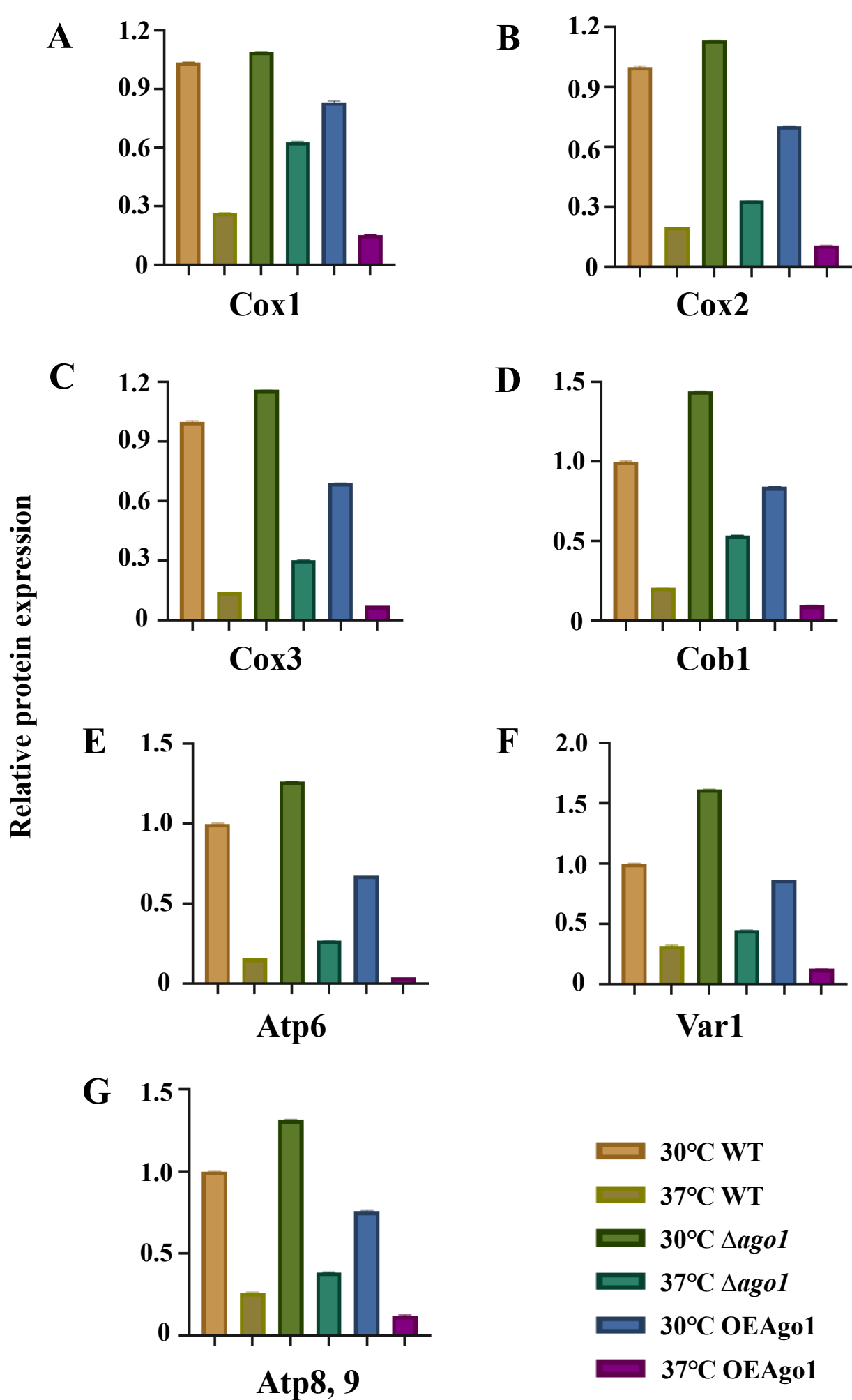

Fig. S2

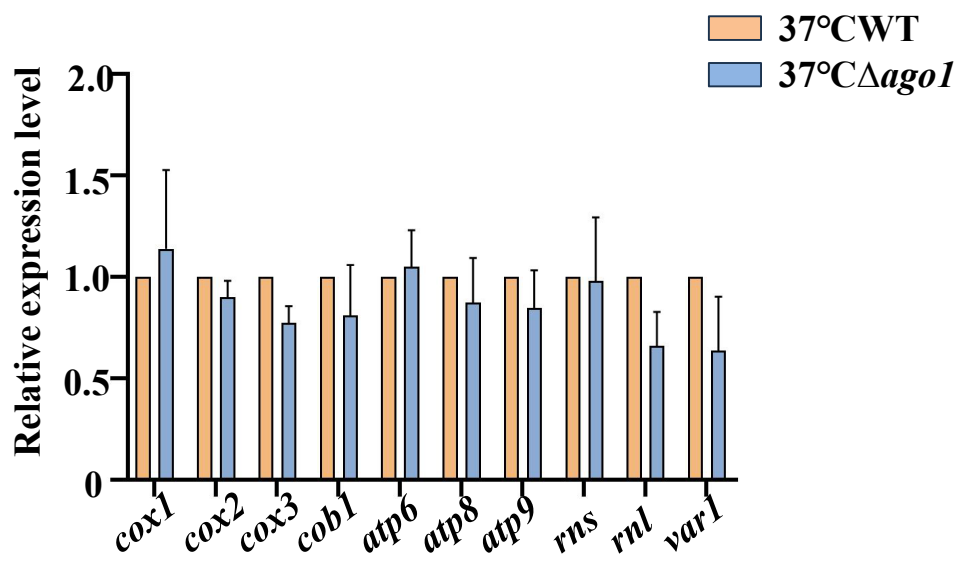

Fig. S3

**A**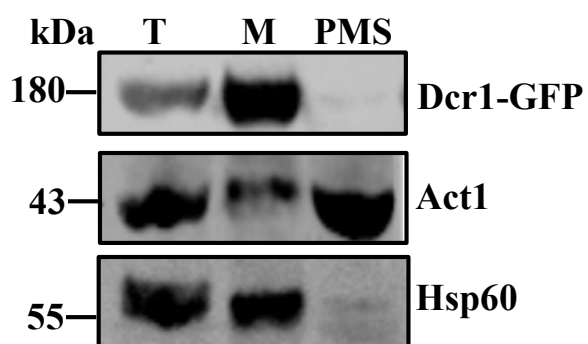**B**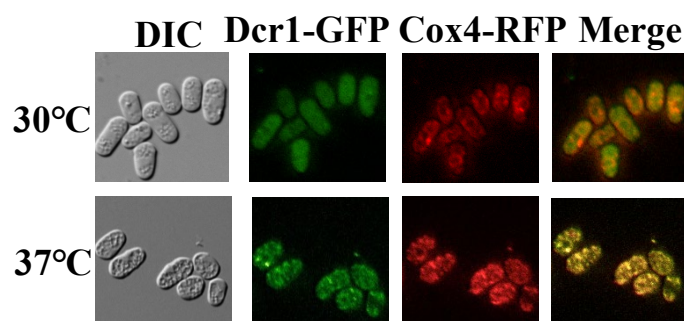

Fig. S4

Supplement: Supplementary Figure 0413 [file mmc3.pdf]
