# Supplementary material for: Ago1 is required for the regulation of mitochondrial translation under heat stress in Schizosaccharomyces pombe
Source: J Biol Chem. 2026 Jun 4;302(7):113235. doi: 10.1016/j.jbc.2026.113235 (PMC13324456; doi:10.1016/j.jbc.2026.113235)
Supplement: Supplementary figure legends [file mmc4.docx]

**Supplementary figure legends**

Fig.S1. Supplementation of *ago1* gene can rescue the mitochondrial protein levels to the levels of WT strains under heat stressed condition. Mitochondrial extracts were prepared from the cells in unstressed and heat stress conditions by spheroplast lysis, and analyzed by western bolting with anti-peptide Abs against mitochondrial-encoded Cob1, Cox1, Cox2, Cox3, Atp6 and Hsp60, as well as nuclear-encoded Cox4, Mrp5, among them, Mrp5 serves as a loading control.

Fig.S2. Quantitative analysis of mitochondrial protein expression levels in *ago1* mutant and overexpression strains under different temperatures. Densitometric quantification of Cox1 (A), Cox2 (B), Cox3 (C), Cob1 (D), Atp6 (E), Varl (F), and Atp8,9 (G) protein levels, normalized to the loading control and expressed as relative protein expression compared to the 30℃ wild-type (WT) group. Data were presented as mean ± SD of independent experiments. Statistically significant differences were determined by Student’s t test (**P* < 0.05, ***P* < 0.01, ****P* < 0.001).

Fig.S3. The qRT-PCR analysis of the Logarithmic-state levels of mature mtRNAs in WT and deleting *ago1* strains with Heat stress treatment (37℃). The strains were cultured in YES medium for 12 h at 37℃. Total RNA was isolated and the mRNA expression levels were measured by qRT-PCR. Levels of mature mtRNAs in 37℃ WT cells were normalized and expressed as fold change over control strain. The mRNA level of each gene was normalized to actin (*act1*) mRNA. The statistical significance was determined by the Student’s t-test using the GraphPad Prism software (**P* < 0.05, ***P* < 0.01, ****P* < 0.001).

Fig.S4. Dcr1 is colocalized in the mitochondria under Heat stress conditions. (A) Dcr1 is localized in mitochondria at the heat stress condition. Mitochondria were isolated from cells expressing Dcr1-GFP as described in Materials and Methods. Total cell extracts (T), mitochondria (M) and postmitochondrial supernatants (PMS) were analyzed by western blotting using anti-GFP Ab to detect Dcr1-GFP, antibody (Ab) against nuclear localized Actin (anti-Actin Ab) and the purified mitochondrial fraction (M) using the antibody against mtDNA-encoded Hsp60 (anti-Hsp60 Ab). (B) Heat stress induces Dcr1 localization into the mitochondria. Cells expressing Dcr1-GFP and Cox4-RFP were grown in Edinburgh Minimal Medium with Leucine deficiency at 30℃ or 37℃. GFP signals were detected by fluorescence microscopy and photographed. Mitochondria was assessed by Cox4-RFP signals. All results were found to be reproducible in at least two experiments.
